# A comparison between two different dried blood substrates in determination of psychoactive substances in postmortem samples

**DOI:** 10.1007/s11419-020-00567-2

**Published:** 2021-01-18

**Authors:** Matteo Moretti, Alessandro Manfredi, Francesca Freni, Carlo Previderé, Antonio Marco Maria Osculati, Pierangela Grignani, Livio Tronconi, Claudia Carelli, Claudia Vignali, Luca Morini

**Affiliations:** 1 Department of Public Health, Experimental and Forensic Medicine, University of Pavia, via Forlanini 12, Pavia, Italy; 2 Legal Medicine Operative Unit, IRCCS Fondazione Mondino, Pavia, Via Mondino 2, 27100 Pavia, PV Italy; 3 Department of Medicine, Surgery and Health, University of Trieste, Piazzale Europa 1, Trieste, Italy

**Keywords:** Flinders Technology Associates card, Dried blood spots, Postmortem, Psychoactive substances

## Abstract

**Purpose:**

Whatman™ 903 cards represent a valid type of support for collection, storage, and analysis of dried blood spots (DBS). Whatman™ FTA (Flinders Technology Associates) are a type of cards soaked in chemicals that cause denaturation of proteins, while preserving DNA and ensuring the safe handling of DBS; to date, these cards are still rarely employed in forensic toxicology. The aim of this study was to analyze several psychoactive substances on not-dried blood on the two different cards and to compare the qualitative and quantitative results.

**Methods:**

Twenty cardiac postmortem blood samples were collected and deposed on Whatman™ 903 and Whatman™ FTA cards. Spots and not-dried blood were analyzed following our validated and previously published liquid chromatography–mass spectrometry methods.

**Results:**

We were able to identify: eight drugs of abuse and their metabolites (15 cases), five benzodiazepines and their metabolites (3 cases), six antidepressants (6 cases) and two antipsychotics (3 cases). We observed a perfect qualitative correspondence and a general good quantitative correlation between data obtained from not-dried blood and the two different DBS cards, except for alprazolam, diazepam, desmethyldiazepam, fluoxetine and sertraline, that showed a lower concentration on FTA. Additional experiments suggest that the chemicals, adsorbed on FTA, are not the cause of the loss of signal observed for the substances previously mentioned and that methanol should be preferred as extraction solvent.

**Conclusions:**

This study proved that FTA cards are a good and a hazard-free alternative sample storage method for analysis of several psychoactive substances in postmortem blood.

**Supplementary Information:**

The online version contains supplementary material available at 10.1007/s11419-020-00567-2.

## Introduction

Dried blood spot (DBS) is a sampling technique that involves the application of very small volumes of blood on a paper-based substrate, which is eventually left to dry; the spots can then subsequently be easily extracted and analyzed. To date, a wide variety of substances has been tested on dried blood samples. The first description of this technique dates back to 1963 by Guthrie et al., who utilized the spots in neonatal screening for congenital phenylketonuria diagnosis [1]. Thanks to its compatibility with a large number of bioanalytical procedures, such as chromatography, mass spectrometry, DNA analysis and enzyme immunoassays, over the years these samples have been studied in several research fields [2]. Among these, the most important were preclinical development of drugs [3], clinical pharmacology [4], therapeutic drug monitoring [5–7], control of environmental contaminants [8], surveillance for microbiological diseases [9], genetics [10] and, more recently, forensic toxicology [11].

Numerous methods, particularly dedicated to the identification and quantification of psychotropic molecules and substances of abuse, have been developed and validated on DBS. Most of these studies focused on Whatman™ 903 cards. This paper-based substrate allows to collect a certain volume of blood (about 75–80 μL per spot) on a fixed surface area (1.27 cm diameter). A good qualitative and quantitative correlation was observed for drugs of abuse, benzodiazepines, antidepressants and antipsychotics measured in fresh blood and in samples dried on Whatman™ 903 cards, collected from postmortem samples. In particular, the analysis of the whole blood spot, rather than of a fixed portion, may limit the variability due to the hematocrit, as well as the different coefficient of diffusion of the monitored substances on the paper substrate [12–14].

The Whatman™ FTA (Flinders Technology Associates) cards represent another valid type of support for the collection, transport, storage and analysis of biological samples. One of the main application areas is genetics [15], as these paper supports are soaked in chemicals that cause lysis of cells, denaturation of proteins and protect nucleic acids from nuclease action, oxidation and UV damage, keeping them intact for future analysis. The papers also rapidly inactivate any virus or organism present in the sample and prevent the growth of microorganisms, ensuring the safe handling of cards without risk of biohazards. This aspect is particularly important nowadays, considering the current emergency linked to the COVID-19 outbreak.

To date, the use of FTA cards in forensic toxicology has been limited and tested on a few classes of psychotropic substances, such as benzodiazepines. The authors spiked 26 analytes among benzodiazepines, z-drugs and metabolites on FTA-Drug Metabolism and Pharmacokinetics (DMPK) cards and observed a high quantitative reliability of the method [16]. Barfield and coauthors developed a robust and sensitive method for the determination of paroxetine in dried plasma spots, using FTA substrate. They concluded that FTA cards can be a reliable alternative matrix to store plasma and whole blood for therapeutic drug monitoring of medicines [17]. However, these two studies are only based on spiked samples, and have not been applied to real positive blood or plasma samples. A comparative study between five different DBS cards for the determination of six immunosuppressants has been performed by Koster and coauthors. The authors observed different performance results depending on the concentration of the analyte investigated [18].

The objective of this study was to compare quantitative and qualitative results obtained from analysis of not-dried postmortem blood samples, and those for blood depositions using two different paper substrates.

## Materials and methods

### Chemicals

Diazepam, desmethyldiazepam, chlordesmethyldiazepam, alprazolam, clonazepam, 7-aminoclonazepam, bromazepam, flurazepam, desalkylflurazepam, midazolam, triazolam, zolpidem, clotiapine, amitriptyline, nortriptyline, haloperidol, fluoxetine, nortriptyline, fluvoxamine, promazine, chlorpromazine, aripiprazole, mirtazapine, desmethylmirtazapine, maprotiline, venlafaxine, desvenlafaxine, sertraline, paliperidone, citalopram, desmethylcitalopram, diazepam-D5, 7-aminoclonazepam-D4 alprazolam-D5, quetiapine-D8, clozapine-D4, and citalopram-D4 methanolic solutions were obtained from Sigma-Aldrich (Milan, Italy); cocaine, cocaethylene (CE), ecgonine methylester (EME), benzoylecgonine (BE), methadone, 2-ethylidene-1,5-dimethyl-3,3-diphenylpyrrolidine (EDDP), morphine, codeine, ketamine, norketamine, amphetamine, methamphetamine, 3,4-methylenedioxymethamphetamine (MDMA), 3,4-methylenedioxy-*N*-ethylamphetamine (MDEA), 1,3-benzodioxolyl-*N*-methylbutanamine (MBDB), 3,4-methylenedioxyamphetamine (MDA), α-pyrrolidinohexiophenone (α-PHP), α-pyrrolidinopentiophenone (α-PVP), cocaine-D3, morphine-D3, methadone-D3, EME-D3, ketamine-D4, MDMA-D4, mephedrone-D5, and methanolic solutions were obtained from Cerilliant (Milan, Italy). Liquid chromatography–mass spectrometry (LC–MS) grade methanol, acetonitrile, formic acid, dichloromethane, 2-propanol and ammonia were purchased from Carlo Erba S.R.L. (Milan, Italy).

The mobile phase consisted of an aqueous solution with 0.1% (v/v) formic acid (A) and acetonitrile with 0.1% (v/v) formic acid (B).

### Instrumentation

Liquid chromatographic tandem mass spectrometric (LC–MS/MS) analyses were performed on an Agilent 1100-1200 Series system (Agilent Technologies, Palo Alto, CA, USA) coupled with a 4000 Q-TRAP (AB SCIEX, Foster City, CA, USA). The LC instrumentation composed of a binary pump, an isocratic pump and an autosampler maintained at room temperature during analysis. The injector needle was externally washed with methanol (3 s) prior to any injection. A Kinetex C18 column (100 × 2.1 mm i.d., 2.6 µm particle size) (Phenomenex, Castelmaggiore, BO, Italy) was kept at 35 °C during the analysis. The chromatographic separation was carried out in reverse phase, with mobile phases consisting of 0.1% (v/v) formic acid in bidistilled water and 0.1% (v/v) formic acid in acetonitrile. The elution was performed in gradient mode and three different elutions were used and were previously published [13–15]. Multiple reaction monitoring (MRM) was optimized using nitrogen as collision gas (with pressure set at level 5) and a dwell time of 30–50 ms. Two transitions for each substance were chosen for identification. To guarantee the best sensitivity, the MRM transitions were divided into two 4 groups. Data acquisition and elaboration were performed by the Analyst^®^ software (version 1.5.1, AB SCIEX).

### Sample analysis protocol

Twenty cardiac blood samples, collected from 20 different autopsy cases which tested positive for at least one psychoactive substance during routine toxicological analyses, were included in the study. Exclusion criteria were: presence of blood clots in the plastic tube (sample not homogeneous); low blood amount (< 10 mL); blood collected from putrefied corpses; and addition of preservatives. After the collection, blood samples were stored in plastic tube at −20 °C until the deposition on the DBS cards. Aliquots of 85 µL of blood were pipetted on the filter cards (three different spots on Whatman™ 903TM and three different spots on Whatman™ FTA, Sigma-Aldrich, Milan, Italy) and left to dry (for about 2 h), keeping them in the dark, at room temperature. Blood spots were analyzed within 24 h from the deposition.

For each spot, the whole blood stain (a disk of about 13 mm diameter) was cut and put into a glass tube, containing 1-mL phosphate buffer solution at pH 6 and all the deuterated internal standards cited above at the concentration of 100 ng/mL. The solutions were sonicated for 10 min, vortexed for 10 s and finally centrifuged at 4000 *g* for 5 min. Supernatant solutions were separated from the filter cards and purified through a Bond Elut Certify I solid-phase extraction (SPE, 200 mg) cartridge (CPS Analitica, Milan, Italy). The cartridges were initially activated with 2-mL methanol, then rinsed with 2-mL phosphate buffer solution at pH 6, before loading samples solutions. The columns were then washed with 2-mL deionized water, 3 mL 0.1 M HCl and finally 5-mL methanol. The analytes elution was carried out with 2-mL dichloromethane–isopropanol mixture (8:2 v/v) with 2% ammonia solution. The eluate solution was dried under nitrogen stream, and reconstituted in 200-µL mobile phase; finally, 5 µL was injected in the LC–MS/MS system. The same procedure was applied also to three aliquots, of 85 µL each, of not-dried blood.

The method for all the analytical procedures has already been validated and previously published [12–14].

### Evaluation of matrix effects, recovery, extraction efficiency and batch stability

Methanolic working solutions containing 45 psychoactive substances (solution A) and internal standards (solution B) were freshly prepared at the concentration of 1000 ng/mL. Twenty microliters of solution A and 20 µL of solution B were added to 1980 µL blank blood and to 1980 µL phosphate buffer solution. Fifty microliters of spiked blood sample were deposed on five FTA cards. The same procedure was applied to spiked buffer solution. The cards were let to dry and, eventually, processed as described in the previous paragraph. Fifty microliters of blank blood samples and blank phosphate buffer solutions were deposed to FTA cards and processed using the same procedure. The solutions A and B were spiked to blank samples after the SPE extraction. Phosphate buffer solutions and blood samples were also analyzed without deposition on FTA cards. All the analyses were carried out in quintuplicate. Batch stability was measured by injecting the same spiked samples at the beginning and at the end of the sequence (about 24 h).

### Correlation between 903 and FTA cards in spiked samples without biological matrix

Due to controversial data obtained for some molecules detected in real samples (issue discussed further below), another experiment was set up during the study. Forty-five psychoactive substances and internal standards were added to a phosphate buffer solution at a final concentration of 1000 ng/mL. Fifty microliters were deposed on FTA and 903 cards and left to dry. The phosphate buffer solution and the cards were then processed, following the sample treatment procedure described above. Twelve different replicates were performed.

## Results

**Fig. 1:**
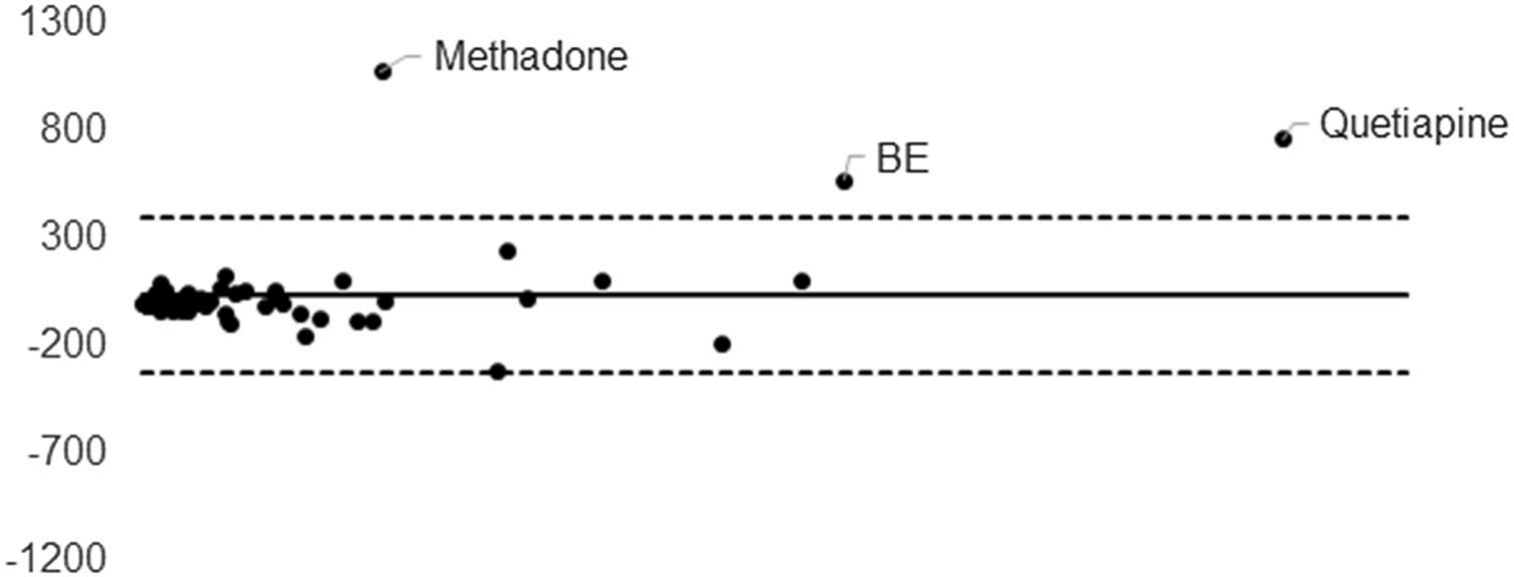
Bland–Altman plot for the results obtained from Whatman™ 903 card, in comparison to the data measured in not-dried blood

**Fig. 2:**
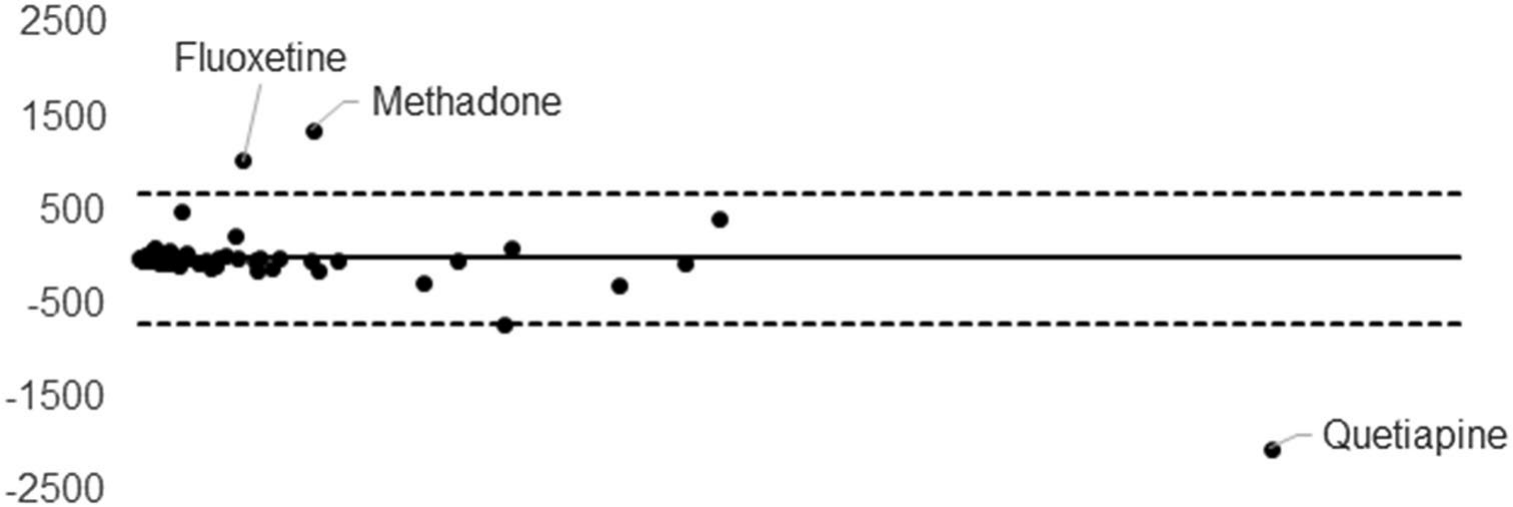
Bland–Altman plot for the results obtained from Whatman™ FTA cards, in comparison to the data measured in not-dried blood

Twenty-one different substances were detected, at least once, among the 20 real blood samples. The identified substances were: cocaine (*n* = 11), BE (*n* = 11), EME (*n* = 11), CE (*n* = 3), morphine (*n* = 4), codeine (*n* = 4), methadone (*n* = 3), EDDP (*n* = 3), 7-amino-clonazepam (*n* = 2), citalopram (*n* = 2), diazepam (*n* = 2), quetiapine (*n* = 2), venlafaxine (*n* = 2), desvenlafaxine (*n* = 2), alprazolam, amitriptyline, bromazepam, desmethyldiazepam, fluoxetine, haloperidol and sertraline. A total of 69 measurements were performed. All the molecules were detected in not-dried blood, in blood dried on classic 903 DBS and in blood dried on FTA cards. The concentrations and the standard deviations are shown in Table 1. Least-squares regression analyses demonstrated a good quantitative correlation between data obtained from not-dried blood and the two different DBS cards, with *r*^2^ = 0.9782 and 0.9405 for 903 and FTA, respectively (*ρ* < 0.005). The Spearman’s coefficient *r*_s_ also confirmed the good correlation, with a calculated value between not-dried blood and 903 DBS of 0.8922 (*ρ* < 0.0001) and between not-dried blood and FTA of 0.8898 (*ρ* < 0.0001). A Bland–Altman plot was set up for the results obtained from the two cards, in comparison to the data measured in not-dried blood. Plots are reported in Figs. 1 and 2. A general good agreement was observed between not-dried blood and the two dried alternatives. Concentrations of fluoxetine and sertraline, measured in FTA0 DBS, were about tenfold lower than those measured in not-dried blood and significantly lower than the ones observed in 903 DBS. A lower concentration in FTA DBS was also observed for some benzodiazepines, namely alprazolam, diazepam and desmethyldiazepam.

**Table 1:** Measured concentrations in not-dried blood, Whatman™ 903 and Whatman™ FTA

Substance	Conc. in not-dried blood (ng/mL)		Conc. in Whatman™ 903 DBS (ng/mL)		Conc. in Whatman™ FTA DBS (ng/mL)	
	Mean	SD	Mean	SD	Mean	SD
Cocaine (*n* = 11)	56.7	2.6	63.5	3.3	62.6	1.8
	302	25.1	295	12.5	295	10.4
	155	11.6	181	24.9	197	34.2
	679	30.7	686	163	670	76.2
	54.7	8.8	46.6	9.6	73.6	11.3
	52.7	5.3	50.3	6.4	57.3	9.2
	40	1.4	50.5	6.7	45.7	4
	231	16.4	231	4	244	13.1
	148	13.7	136	13.5	158	13.3
	1350	40	1340	65.6	1357	92.9
	85.3	3.1	71	5.8	70.1	19.6
BE (*n* = 11)	443	32.6	520	37.3	517	12.3
	1170	45.1	1060	104	1180	81.9
	745	23.1	718	27.6	873	13.8
	2140	217	1900	28.6	2830	705
	1150	1230	1230	20.8	1280	145
	441	27.9	541	17.9	526	15.3
	87.1	14	117	16.8	105	5.8
	771	27.8	709	13.9	792	17.6
	948	49.2	1010	108	943	49.8
	4170	270	3600	130	3750	261
	3700	431	3590	431	3750	329
EME (*n* = 11)	192	6.4	178	25.5	226	24.9
	608	7.8	543	56	564	23.8
	381	21.2	364	28.5	427	69.9
	2150	70	2130	172	2180	197
	476	22	403	61.8	556	55.3
	108	3.6	108	9	148	8.4
	52	3.8	59.6	8.7	64.4	3.7
	439	10.5	477	18.2	456	47.9
	335	7.8	307	6.6	335	8.6
	3120	138	3310	221	3410	85
	846	30	894	40.3	952	37.7
CE (*n* = 3)	103	4.3	102	12.6	107	6.4
	104	11.5	96.6	10.2	108	12.7
	81.1	5.2	76	4.4	70.7	6.3
Morphine (*n* = 4)	34.4	3.3	34.6	0.7	33.6	3.4
	206	7.5	240	18	208	32
	155	4.6	98.9	8.1	149	29.1
	237	11.5	267	41.6	299	23.1
Codeine (*n* = 4)	19.2	1.2	19.3	1.5	20.4	1.3
	27.5	0.8	31.8	2.3	31.9	1.3
	21.8	1.5	22.2	1.1	27.3	6.6
	31.9	0.4	34.6	2.4	31.5	3.1
Methadone (*n* = 3)	781	231	781	91.1	527	147
	1870	1960	785	33	499	12.5
	241	13	264	43.1	162	23.2
EDDP (*n* = 3)	102	13.2	56.9	6.2	50.7	13.6
	158	169	60.2	2.5	49.4	2.5
	35.9	1.6	45.2	9.3	38.6	2.8
Quetiapine (*n* = 2)	276	30.5	223	16.5	268	44
	6700	1060	5930	621	8730	1710
Citalopram (*n* = 2)	11.1	0.2	9.8	1.5	6.5	0.9
	345	34.7	359	14.5	295	24
Venlafaxine (*n* = 2)	1810	233	2120	537	2070	548
	2590	50.3	2490	17.7	2480	444
Desvenlafaxine (*n* = 2)	541	82.1	486	15	545	25.8
	832	73.4	976	329	819	82
Diazepam (*n* = 2)	18.5	2	19.3	2.4	6.3	1.1
	58.1	3.6	43.7	5.3	23.6	5.2
7-Amino-clonazepam (*n* = 2)	33.9	4.4	29.8	3.3	41.4	2.5
	82.7	4.5	72.5	1.4	85.2	12.2
Alprazolam (*n* = 1)	37.8	2.9	34.6	4.9	18.9	2.5
Amitriptyline (*n* = 1)	66.7	10.9	56.3	4.3	42.1	9.7
Bromazepam (*n* = 1)	247	15.7	220	24.1	217	15.6
Desmethyldiazepam (*n* = 1)	157	5.7	145	15.3	44.4	6.9
Fluoxetine (*n* = 1)	1230	94.5	1310	95.4	185	67
Haloperidol (*n* = 1)	7	0.2	7.9	0.8	6.5	0.9
Sertraline (*n* = 1)	530	106	395	31.6	28.1	2

Matrix effects, recovery and extraction efficiency were considered acceptable for all the substances, except for amitriptyline, venlafaxine, desvenlafaxine and codeine. All the results are reported as supplementary material (Table S1). Extraction efficiency was measured by comparing the signals obtained from spiked blood samples deposed on FTA cards and spiked bloods samples directly processed. Recovery was reported as the percentage of standard solutions spiked on DBS cards before SPE extraction with same samples spiked at the same concentration after SPE procedure.

In particular, matrix effects were considered negligible for almost all the substances, except for amphetamine, methamphetamine, MDMA, MBDB and chlorpromazine, for which a slight ion suppression was observed. A general high recovery percentage was observed for most of the drugs of abuse. On the contrary, low recoveries were measured for most of benzodiazepines. The lower p*K*_a_ of these molecules, together with their chemical characteristics represent the main reason for a low recovery. However, the SPE procedure guarantees a good repeatability for those substances, as already demonstrated in a previous paper [13]. All the monitored molecules were stable within the sequence run (about 24 h).

**Fig. 3:**
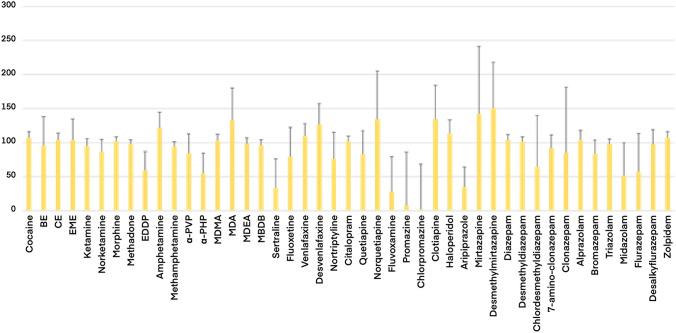
903/buffer solution peak area ratios at fixed concentration (100 ng/mL)

**Fig. 4:**
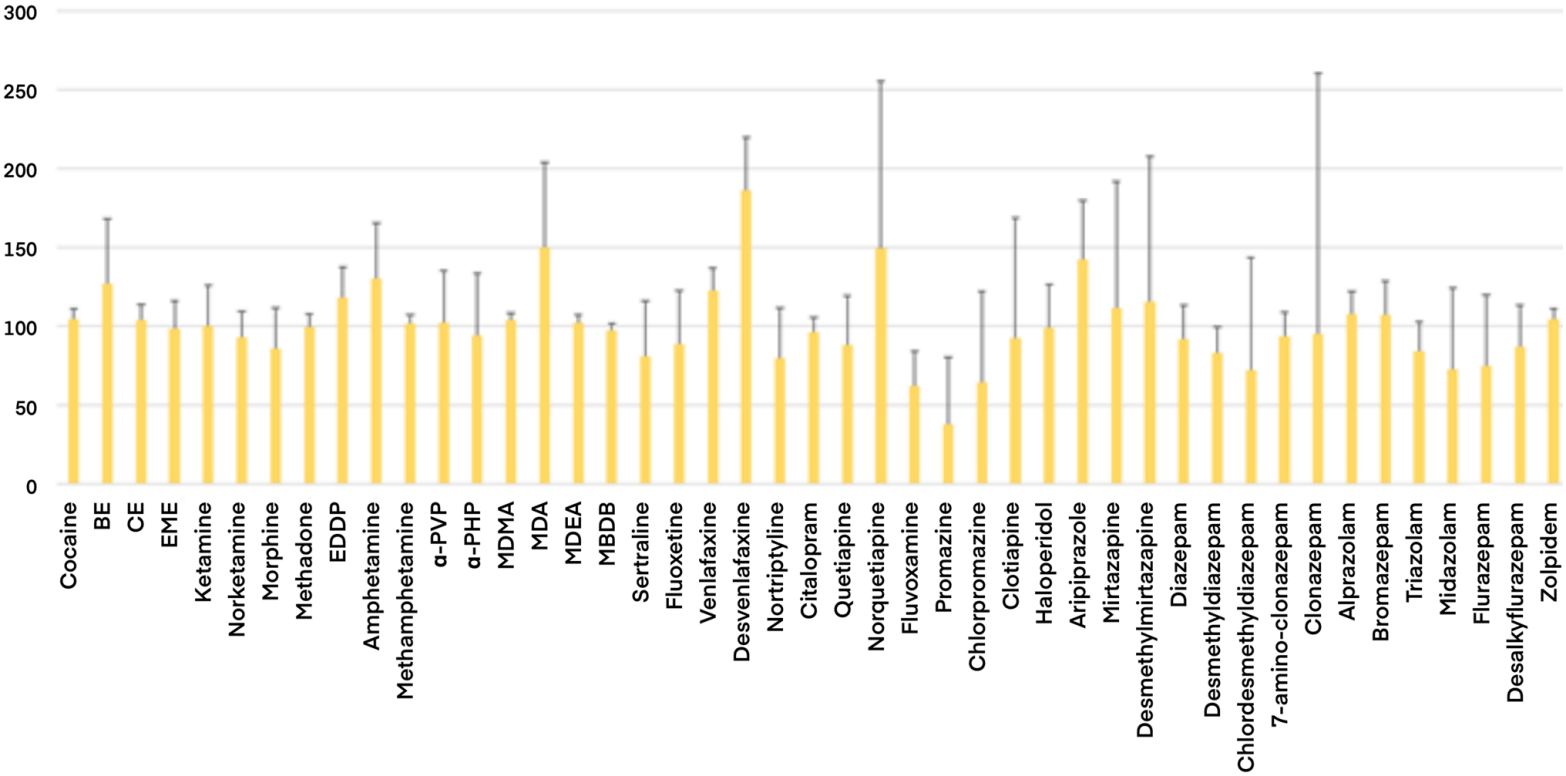
FTA/buffer solution peak area ratios at fixed concentration (100 ng/mL)

To evaluate a potential degradation of these molecules after deposition on the FTA (e.g., due to chemicals adsorbed in the card), a further experiment was performed. Fifty microliters phosphate buffer solution containing all the 21 substances detected in real samples, together with other 24 psychoactive substances with different physical and chemical characteristics, were deposed on the two different DBS cards (*n* = 12 replicates for each), left to dry, processed as described above, and matched to data obtained from the analyses of a same volume of not-dried phosphate buffer solution. The peak areas of each substance, obtained from all the 903 and FTA samples, after normalization by proper internal standards, were compared with those ones obtained from the standard solutions. The 903/buffer solution and FTA/ buffer solution ratios are reported in Figs. 3 and 4, respectively. The peak area ratios were generally in better agreement between FTA DBS and phosphate buffer solution rather than those measured in samples deposed on 903 DBS. Yet, the massive loss of signal measured for fluoxetine and sertraline in real dried blood samples, was not observed in spiked phosphate buffer solution dried on the same FTA cards.

## Discussion

This study was performed to compare the qualitative and quantitative results obtained from blood stored under routine conditions, and blood dried on two different DBS cards. In particular, previous studies on postmortem samples, performed on Whatman™ 903 substrates, demonstrated that the data were in good agreement with the ones carried out in not-dried blood [12–14]. However, FTA cards differ from 903 ones because of the chemicals adsorbed on the paper substrate, that allow to lyse cells and denature proteins, while protecting nucleic acids from nuclease action, UV, and oxidative damage. This factor may influence the stability of some molecules of forensic interest, such as psychoactive substances.

Preliminary data, obtained from 20 positive postmortem samples, confirmed that 903 cards provide similar results in terms of identification and quantification of the monitored substances. In particular, <10% of the data obtained from the 903 DBS yielded a concentration lower than 85% respect to that measured in blood stored at routine conditions. A similar good agreement was also assessed by the response of the Bland–Altman plot and the Spearman’s rho value. Moreover, a general good correlation was observed between data, carried out through the analyses of blood samples, deposed on FTA cards, and analyses of not-dried blood. Cocaine and its main metabolites were the most frequently detected substances. Eleven out of 20 cases were positive for cocaine, BE and EME. The comparison between concentrations measured in stored blood and samples dried on cards confirmed previously published data, and proved that DBS are a good alternative sample storage, independently on the paper substrate type. Similar results were obtained for morphine and codeine. On the contrary, one case for methadone and two cases for 2-ethylidene-1,5-dimethyl-3,3-diphenylpyrrolidine (EDDP) provided lower concentrations in cards compared to the ones measured in not-dried blood. Most of the antidepressants yielded similar quantitative results in routinely stored blood and in blood dried on the two different cards. However, an almost complete degradation was reported for two antidepressants, namely sertraline and fluoxetine. Moreover, also results obtained from the quantitative determination of alprazolam, diazepam and desmethyldiazepam in blood samples, dried on FTA substrate, were significantly lower than those measured in blood stored at routine conditions. Initially, it was hypothesized that chemicals adsorbed on the FTA could have influenced the stability of these compounds: a further experiment, without biological matrix, was, therefore, set up, to assess this hypothesis. Forty-five psychoactive substances, including all those ones detected in real samples, were diluted in a phosphate buffer solution at a final concentration of 1000 ng/mL. Eventually, 50 µL of the solution was deposed on 12 different spots of FTA and 12 spots of 903 filter papers. The 12 samples were measured and compared to 12 samples of buffer solution at the same volume and concentration. The results from this experiment, reported in Figs. 3 and 4, were clearly in contrast with those obtained from real postmortem samples. Indeed, a good correlation between peak area ratios, measured in solution, and in samples deposed on FTA substrate, was observed for most of the compounds, and was generally higher than the correlation between not-dried buffer solution and samples dried on 903 cards. Moreover, the standard deviations, measured for FTA samples, were lower than the ones calculated for samples deposed on 903 papers. The experiments on standard solutions highlighted a degradation of phenothiazines on DBS cards, independently on the paper substrate. In fact, an almost complete loss of promazine was observed in 903 card, together with a significant decrease of the signal of chlorpromazine. A more limited loss was achieved using the FTA cards. However, preliminary data on standard solutions suggested that the quantitative determination of phenothiazines in samples deposed in DBS should be interpreted with caution.

The results of the experiment with phosphate standard solutions confirmed that chemicals adsorbed on the FTA card are not the cause of the loss of signal observed for sertraline, fluoxetine and the three benzodiazepines. Therefore, we supposed that the differences between data obtained from real samples and standard solutions concern the biological matrix. We deposed other aliquots of blood samples, positive for sertraline, fluoxetine and diazepam (three aliquots for each sample) on FTA and 903 cards. The extraction was performed by adding 1 mL methanol, instead of 1 mL phosphate buffer. Two microliters of buffer solution were added to the samples just before SPE procedure. The rest of sample preparation was the same as the one described above. The analytical data were compared to the ones obtained from blood samples stored at routine conditions, and extracted with 1 mL methanol. The differences between peak area ratios measured in blood samples dried on FTA and extracted with methanol were not significantly different (<15% standard deviations) from the ones measured in blood samples deposed on 903 cards and in not-dried blood samples. These results suggested that methanol should be preferred as extraction solvent, when FTA cards are used.

## Conclusion

This study proved that FTA DBS cards are a good and a hazard-free alternative sample storage for analysis of most of the psychoactive substances detected in postmortem blood. Preliminary data, obtained from 20 real cases, confirmed a good qualitative and quantitative agreement between concentrations of different drugs of abuse, antidepressants, antipsychotics, benzodiazepines and metabolites, measured in blood stored at routine conditions, and blood dried on DBS cards, independently on the type of paper substrate, except for diazepam, desmethyldiazepam, alprazolam, fluoxetine and sertraline, that provided a lower extraction from FTA cards. Phosphate buffer solution did not provide a high extraction efficiency, when applied to FTA cards, for all the substances detected. Methanol seemed to increase the extraction performance, and therefore, should be preferred whenever FTA cards are used.

## Supplementary Information

Below is the link to the electronic supplementary material.

Supplementary file1 (DOCX 18 KB)
