# Supplementary material for: A comparison between two different dried blood substrates in determination of psychoactive substances in postmortem samples
Source: Forensic Toxicol. 2021 Jan 18;39(2):385–93. doi: 10.1007/s11419-020-00567-2 (PMC7812343; doi:10.1007/s11419-020-00567-2)
Supplement: Supplementary file 1 — Supplementary file1 (DOCX 18 KB) [file 11419_2020_567_MOESM1_ESM.docx]

| Substance | Extraction efficiency  (%) | Recovery  (%) | Matrix effect  (%) |
| --- | --- | --- | --- |
| Cocaine | 70.7 | 95.1 | 116.1 |
| BE | 87.6 | 69.1 | 115.7 |
| EME | 66.2 | 67.7 | 101.7 |
| CE | 64.6 | 88.1 | 97.1 |
| Morphine | 75.2 | 53.2 | 112 |
| Methadone | 75.9 | 44.9 | 91 |
| EDDP | 61.8 | 55.2 | 96.6 |
| Amphetamine | 76.4 | 55.9 | 75.2 |
| Methamphetamine | 79.2 | 51.7 | 74.6 |
| MDMA | 69.4 | 84.7 | 71.6 |
| MDEA | 72.8 | 48.8 | 120 |
| MDA | 64.9 | 57.3 | 115 |
| MBDB | 74.9 | 48.4 | 76.5 |
| Ketamine | 83 | 76.4 | 110 |
| Norketamine | 71.7 | 107.9 | 101.6 |
| α-PVP | 97.9 | 89.3 | 113 |
| α-PHP | 88.3 | 90.5 | 126.9 |
| Alprazolam | 60.1 | 35.8 | 123.8 |
| Bromazepam | 72.7 | 55.6 | 109.5 |
| Chlordesmethyldiazepam | 69.1 | 22.2 | 111.6 |
| Clonazepam | 95 | 29.3 | 108.7 |
| 7-amino-clonazepam | 81.4 | 38.2 | 90.2 |
| Diazepam | 71.6 | 33.6 | 107.7 |
| Desmethyldiazepam | 63.8 | 32.5 | 118.4 |
| Flurazepam | 62.7 | 56.3 | 114 |
| Desalkylflurazepam | 60.3 | 34.8 | 110.1 |
| Midazolam | 93.8 | 45.3 | 114.3 |
| Triazolam | 68.9 | 41.6 | 109.9 |
| Zolpidem | 81.1 | 105.2 | 115.1 |
| Aripiprazole | 75.8 | 65 | 106.1 |
| Chlorpromazine | 109.8 | 36.7 | 70.1 |
| Citalopram | 73.7 | 28.5 | 119.4 |
| Clothiapine | 112 | 49 | 109.9 |
| Fluoxetine | 77.8 | 90.3 | 87.3 |
| Fluvoxamine | 83.3 | 19 | 112.2 |
| Haloperidol | 71.1 | 51.5 | 104.5 |
| Mirtazapine | 65.1 | 48.6 | 113 |
| Desmethylmirtazapine | 113.7 | 45.3 | 105.1 |
| Nortriptiline | 67.8 | 57.9 | 94 |
| Promazine | 109.8 | 28.4 | 108.6 |
| Quetiapine | 86.5 | 41 | 94.1 |
| Norquetiapine | 93.5 | 30 | 113.7 |
| Sertraline | 69.8 | 32 | 80 |

**Table S1** Extraction efficiency, recovery and matrix effects measured at 200 ng/mL on FTA cards.
